# Cross-cultural adaptation, translation and pre-testing of the Caregiver Analysis of Reported Experiences with Swallowing Disorders (CARES) screening tool in Kannada

**DOI:** 10.1186/s41687-025-00863-8

**Published:** 2025-09-01

**Authors:** Adithi G. Hegde, Srikanth Nayak, R. Vani Lakshmi, Krishna Sharan, Janet Jaison Varghese, Usha Devadas

**Affiliations:** 1 Department of Speech and Hearing, Manipal College of Health Professions, Manipal Academy of Higher Education, Manipal, Karnataka 576 104 India; 2 Department of Audiology and Speech-Language Pathology, Yenepoya Medical College, Yenepoya University (Deemed to be University), Mangalore, Karnataka India; 3 Department of Data Science, Prasanna College of Public Health, Manipal Academy of Higher Education, Manipal, Karnataka India; 4 Department of Radiotherapy and Oncology, Kasturba Medical College, Manipal Academy of Higher Education, Manipal, Karnataka India

**Keywords:** Screening tool, Dysphagia, Head and neck cancer, Caregiver burden, Quality of life

## Abstract

**Background:**

Dysphagia in a family member may negatively influence caregivers and may disrupt their quality of life. Thus, it is crucial to understand caregiver perspectives as an integral component of holistic dysphagia treatment. Hence, the present study aimed to cross-culturally adapt, translate, and pre-test the Caregiver’s Analysis of Reported Experiences with Swallowing Disorders (CARES) into the Kannada language, a structural screening tool for assessing caregiver burden associated with dysphagia.

**Methods:**

The Kannada version of the CARES screening tool was cross-culturally adapted using the ISPOR Principles of Good Practice for Patient-Reported Outcome Measures and was pre-tested on 48 dysphagia caregivers of adult dysphagia cancer survivors. The internal consistency of each item was assessed using Cronbach’s alpha.

**Results:**

The results revealed that the Kannada version of the CARES screening tool had good internal consistency for each subscale, Part A—Checklist of Behavioral and Functional Changes”, and Part B—Measures of Subjective Caregiver Stress, with α = 0.763 and α = 0.852, respectively. The item-total correlation of each item of the subscale was greater than 0.8, indicating that each item of the tool significantly contributed to the tool.

**Conclusions:**

The Kannada CARES was found to have good internal consistency. Hence, it can be considered a linguistically equivalent tool for identifying caregiver burdens associated with dysphagia care.

**Supplementary Information:**

The online version contains supplementary material available at 10.1186/s41687-025-00863-8.

## Introduction

Swallowing disorders or dysphagia are the consequence of impairments in the structure and function of the oral cavity, larynx, pharynx, esophagus, or gastroesophageal junction [1, 2]. Dysphagia is a burdensome condition affecting both the aging population and individuals with neurological disorders such as stroke, Parkinson’s disease, dementia, and head and neck cancer [3–5]. The biological sequelae of dysphagia include dehydration, aspiration pneumonia, malnutrition, and an overall decline in general health. The impact of dysphagia extends beyond individuals with the disorder and places a significant burden on their caregivers [6]. Caregiver’s burden refers to the subjective physical, emotional, psychological, and economic burden experienced by caregivers who care for individuals with dysphagia [7, 8]. Caregiver’s burden is associated with various concerns, such as fear of choking, limited food choices, nutritional concerns, and grief and acceptance of their lives as the “new normal” [9–11].

Studies have shown that caregivers have elevated levels of emotional burden when their care receiver has dysphagia, with 70% grading their burden as moderate to severe [12]. Adult caregivers of parents with swallowing disorders experience emotional burdens that are 1.61 times greater than those of parents without swallowing difficulties [12]. Additionally, individuals caring for a family member with dysphagia experience a range of other issues, such as disruptions in socializing [12], changes in routine, and going out together [13]. Caring for someone with dysphagia can lead to a “third-party disability,” where the caregivers themselves experience reduced quality of life and issues stemming from providing dysphagia care [9, 14]. Therefore, reducing the burden on caregivers should be a vital aspect of an effective dysphagia management strategy.

To address caregiver burden effectively, tools that can assess the challenges faced by dysphagia caregivers are crucial. Hence, it is essential to develop effective tools to address caregivers’ burdens and assess the challenges they face. However, such tools are scarce in the literature [15, 16]. Considering the need to understand how dysphagia independently burdens caregivers, Shune et al. [17] developed the “Caregiver Analysis of Reported Experience with Swallowing Disorders (CARES)” tool to screen the burden encountered by the caregivers of recipients with dysphagia in English-speaking populations. The CARES questionnaire has 26 items, 10 of which measure behavioral and functional changes, whereas the remaining 16 measure caregiver stress. These items assess caregivers’ practical challenges, such as monitoring their nutritional status, financial and occupational impact, quality of life, coping strategies, and impact on self-well-being. At the end of each subscale, the caregivers are asked to identify one statement that they find the most burdensome compared with others. The CARES has shown promising results in quantifying the burden experienced by caregivers in English-speaking populations caring for individuals with dysphagia, with good internal consistency overall (Cronbach’s alpha values of 0.79) and for Part B (Cronbach’s alpha values of 0.77), whereas values for Part A were slightly lower (Cronbach’s alpha values of 0.65) [17]. Furthermore, the authors reported a positive association between CARES scores and EAT-10 scores (*r* = 0.72), indicating an increase in caregiver burden with increasing severity of dysphagia in care recipients [17].

Understanding caregiver burden is essential for the holistic management of adults with dysphagia, and CARES is a tool that can be used to quantify caregiver burden. However, adapting and validating such tools in other languages is necessary to gain insight into caregiver burden and provide appropriate guidance for comprehensive dysphagia management. Unfortunately, there are no such tools available in any Indian languages at present. Therefore, we aim to address this scarcity by adapting and culturally validating the tool for clinical and research use among the Kannada-speaking population who require dysphagia rehabilitation services. The primary aim of our study was to culturally adapt, translate, and pre-test the English version of the CARES into Kannada. The secondary objective was to identify which components caregivers find most burdensome on the basis of their CARES responses.

## Method

### Development of the Kannada CARES

**Fig. 1:**
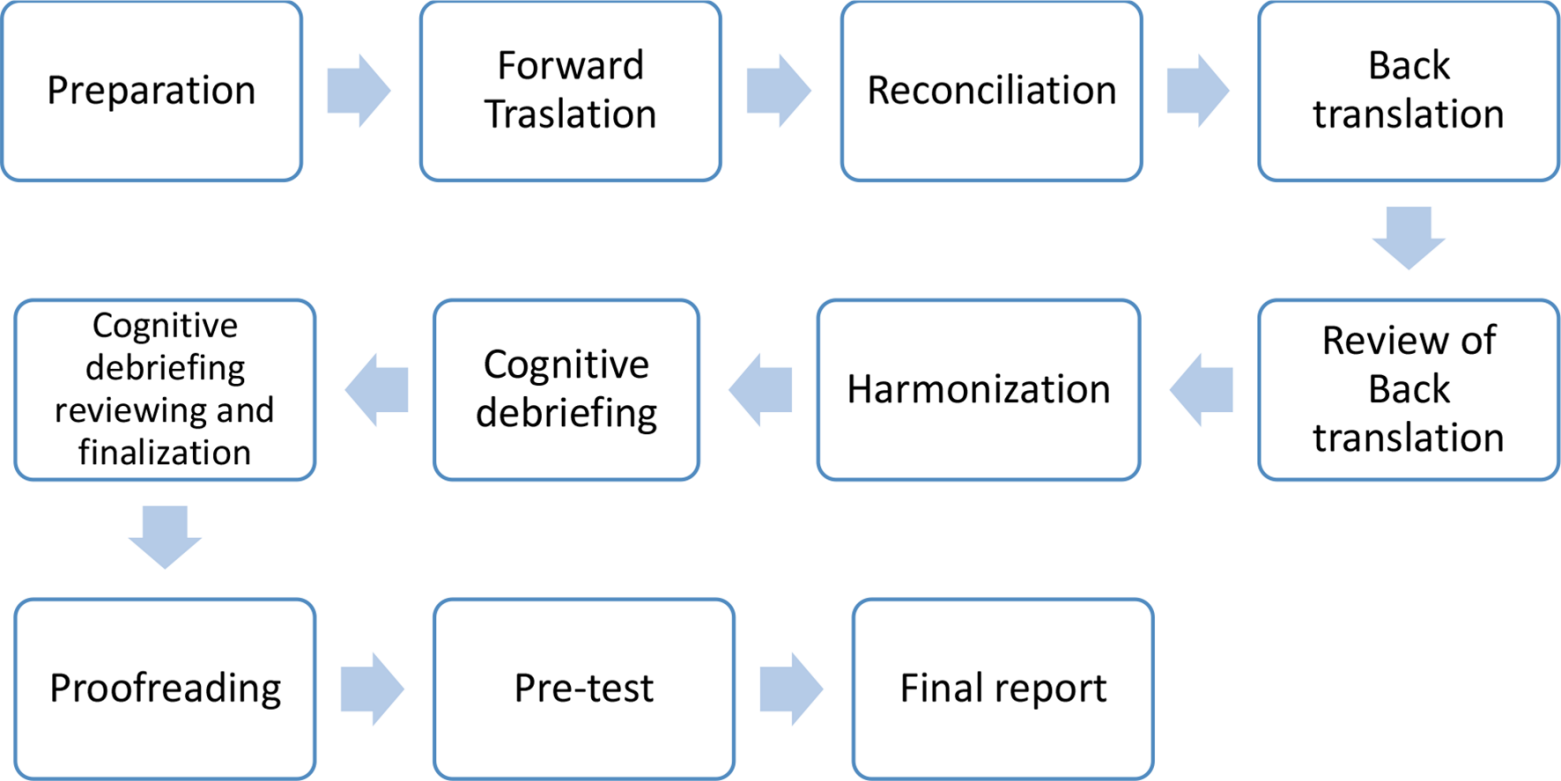
Flowchart of the translation process of the CARES screening tool in Kannada

The study was approved by the Institutional Ethical Committee (IEC), Kasturba Medical College (IEC: 328/2022), Manipal Academy of Higher Education, India. Permission was obtained from the developer of CARES to translate and culturally adapt the CARES screening tool to the Kannada language. The Kannada version of the CARES screening tool was translated and culturally adapted for the present study following the guidelines of the “ISPOR Principles of Good Practice for the Cross-Cultural Adaptation Process for Patient-Reported Outcomes Measures” [18]. A flowchart of the translation and adaptation process is depicted in Fig. 1.

***Step 1*—*Preparation***.

- Prior permission was obtained from the developer of CARES to translate, adapt, and pre-test the CARES screening tool to the Kannada language.
- The developer of CARES was invited to participate in the translation and cultural adaptation process of the Kannada CARES version.
- Two Speech-language pathologists (SLPs) and one non-SLP proficient in Kannada and English were identified for forward and backward translation.

#### Step 2—Forward translation

- Two individuals fluent in English and the Kannada language, an SLP and a non-SLP, were identified and requested to participate in the forward translation of the questionnaire. Prior to forward translation, they were asked to evaluate the sociocultural suitability of the questionnaire in terms of the relevance of the questionnaire content to Indian cultural values, caregiving practices, and patient demographics to ensure that the questionnaire content was culturally appropriate and meaningful for the target population to avoid misinterpretations during translation.
- Further, these two translators were explained about the purpose of the questionnaire to ensure that the conceptual meaning was maintained during the translation process. By carefully addressing each item in the questionnaire for cultural relevance, the translators translated the questionnaire into the Kannada language, which resonates with caregivers’ experiences and needs.
- As part of the cross-cultural adaptation process, the items were modified to align with Indian cultural norms and language usage, including adapting terminologies and context-specific references to be culturally appropriate and relevant for Kannada-speaking caregivers.

#### Step 3—Reconciliation

The researchers carefully analyzed the two forward-translated questionnaires and synthesized them into a single forward translation by selecting the most appropriate statements. The single synthesized forward translation was discussed with both translators to identify any discrepancies or areas that require any modifications and to enhance the accuracy and cultural relevance of the forward translated version.

#### Step 4—Back translation

The synthesized Kannada forward translation was provided to two additional translators (SLPs) proficient in both the Kannada and English languages for back-translation into the English language, who were blinded to the original English CARES.

#### Step 5 — Review of back translation

The researchers carefully analyzed the two backward-translated questionnaires and synthesized them into a single backward translation by selecting the most appropriate statements in comparison with the original English questionnaire in terms of content and meaning. The single synthesized backward translation was discussed with both translators to identify any discrepancies or areas that require any modifications and to enhance the accuracy and cultural relevance of the backward translated version.

#### Step 6 — Harmonization

The single synthesized back-translated questionnaire was shared with the developer of the CARES tool for approval and suggestions. The discrepancies that the author noted were considered, and the most culturally appropriate words were included with the input of an SLP proficient in the Kannada language.

#### Step 7—Cognitive debriefing

Further, the Kannada version of the CARES tool was given to two English-Kannada bilingual speech-language pathologists, an oncologist, and a caregiver to determine the sociocultural appropriateness (semantic, idiomatic, and experiential equivalence) of the tool.

#### Step 8— cognitive debriefing review and finalization

The feedback and recommendations received from two SLPs, one oncologist and one caregiver, were analyzed, and necessary modifications were integrated into the tool. The tool was then tested on the five caregivers for face validity assessment. The caregivers were asked to review each item in the questionnaire and provide feedback on its clarity, specifically noting any questions that were unclear or confusing.

#### Step 9 —Proofreading

Furthermore, if any typographical or grammatical errors were corrected by careful proofreading by an SLP proficient in the Kannada language, and a final draft of Kannada CARES was developed. The Kannada CARES Tool included all 26 items of the English version (with cultural modification) divided into two subscales: Part A, “Checklist of Behavioral and Functional Changes,” with 10 items, and Part B, “Measures of Subjective Caregiver Stress,” with 16 items along with a question at the end of each section to identify the most burdensome item. Each item was scored as “YES” or “NO,” with a maximum score of 10 for Part A and 16 for Part B. A higher score indicated a greater burden experienced by the caregivers.

#### Step 10— final report

A description of the process with modifications and translation decisions performed in each step was documented.

### Pre-testing of the final version of CARES-Kannada

Pre-testing of the final version of the translated questionnaire was carried out as a cross-sectional study. The sample size was computed as per the requirements of the minimum number of samples required to estimate Cronbach’s alpha. The minimum sample size required for the study, with an anticipated Cronbach’s alpha of 0.8 and a precision of 0.1 at the 5% significance level for a tool involving 26 items with an expected dropout of 20%, was 48 participants.

The participants were caregivers of head and neck cancer patients. These patients were referred from the Department of Radiation Oncology to the Department of Speech and Hearing at Kasturba Hospital, a tertiary care center in Manipal, India, for swallowing assessments. The study included primary caregivers aged 18 years and above who were proficient in reading and writing Kannada. The caregivers responsible for providing direct care to individuals with head and neck cancer experiencing dysphagia, specifically managing their feeding and nutritional needs, were included. To ensure data independence, only one primary caregiver was included for each care recipient in the study. The primary caregivers of individuals with dysphagia were contacted during the hospital visit, and the purpose of the study was explained. Only the eligible care givers who consented to participate were included in the study. Caregivers who relied on home nursing staff for assistance were excluded. Care recipients or patients with dysphagia were required to undergo active treatment or receive follow-up care at the time of the study. The Mann Assessment of Swallowing Ability-Cancer (MASA-C) [19] was used to assess the severity of dysphagia in the care recipients, which is specifically developed to assess the severity of dysphagia in patients with head and neck cancer. A self-report questionnaire was used to gather the following information on the caregivers: their relationship with the recipients, employment status, education history, hours spent on caregiving, family dynamics, and medical history (Appendix A). The caregivers were subsequently asked to complete the Kannada CARES screening tool. Furthermore, to assess test-retest reliability, a subset of 10 caregivers were randomly selected and asked to complete the CARES screening tool within one week of their initial response.

### Statistical analysis

The data analysis was performed via Jamovi (version 2.3) [20]. Descriptive statistics (means, standard deviations, and percentages) were used to describe the demographic information pertaining to the caregivers and the care recipients. The internal consistency of each item was assessed via Cronbach’s alpha. Test-retest reliability was measured via the intraclass correlation coefficient (ICC). The normality of the data was tested using the Shapiro‒Wilk test, and as the data were found to be normally distributed, Pearson’s correlation coefficient was calculated to assess the relationship between CARES scores and MASA-C scores.

## Results

### Socio-cultural adaptation

In the process of cultural adaptation conducted by two independent bilingual individuals, two items in the original questionnaire were deemed inappropriate for the Indian context and subsequently modified. The original version, along with the reasons for the modifications, are detailed in Table 1.

**Table 1:** Details of cross-cultural modifications performed during forward translation

Original CARES items	Cultural modifications done
**Part A**	
3. Because of my loved one’s swallowing difficulties, the costs associated with their nutrition-related needs have increased (e.g., supplies for tube feeding, thickening products or thickened liquids, and supplements).	The word “thickening products” was replaced with “thicker beverages” in the statement to make it culturally appropriate for the Indian population.
**Part B**	
1. I do not feel prepared to help manage my loved one’s swallowing difficulty (e.g., related to tube feeding, thickened liquids, Heimlich).	The Kannada CARES manual does not include the term ‘Heimlich’ as the maneuver is unfamiliar to the Indian population. Even though caregivers in India use other methods to relieve an individual from airway blockage (e.g., asking the person to look upward and/or tapping on the head), these alternative maneuvers differ significantly from the Heimlich maneuver.

#### Harmonization of the back-translated versions

The few discrepancies and subsequent modifications that took place during this stage are explained in detail as follows: In Q4 of Part B, the word “choke” was initially translated as “/usiru kaʈʈu/”, which was backtranslated to “breathing difficulty”. Hence, the developer of CARES suggested changing the word to better capture the term “obstructed airway” or “food blocking the airway.” Hence, the Kannada translation was changed to “/aha: raʋu sʋa: sana:ɭad̪alli/ /sikkikoɭɭuʋud̪u/,” indicating “food blocking the airway.” In accordance with the suggestions of the developer of CARES, the researchers identified the most pertinent questions in the Kannada version and retained them for finalization.

#### Cognitive debriefing review and finalization

A panel comprising experts and caregivers verified the semantic, idiomatic, experiential, and conceptual consistency across the original and translated versions of the questionnaire items. Table 2 outlines the cultural adjustments suggested by the panel.

**Table 2:**
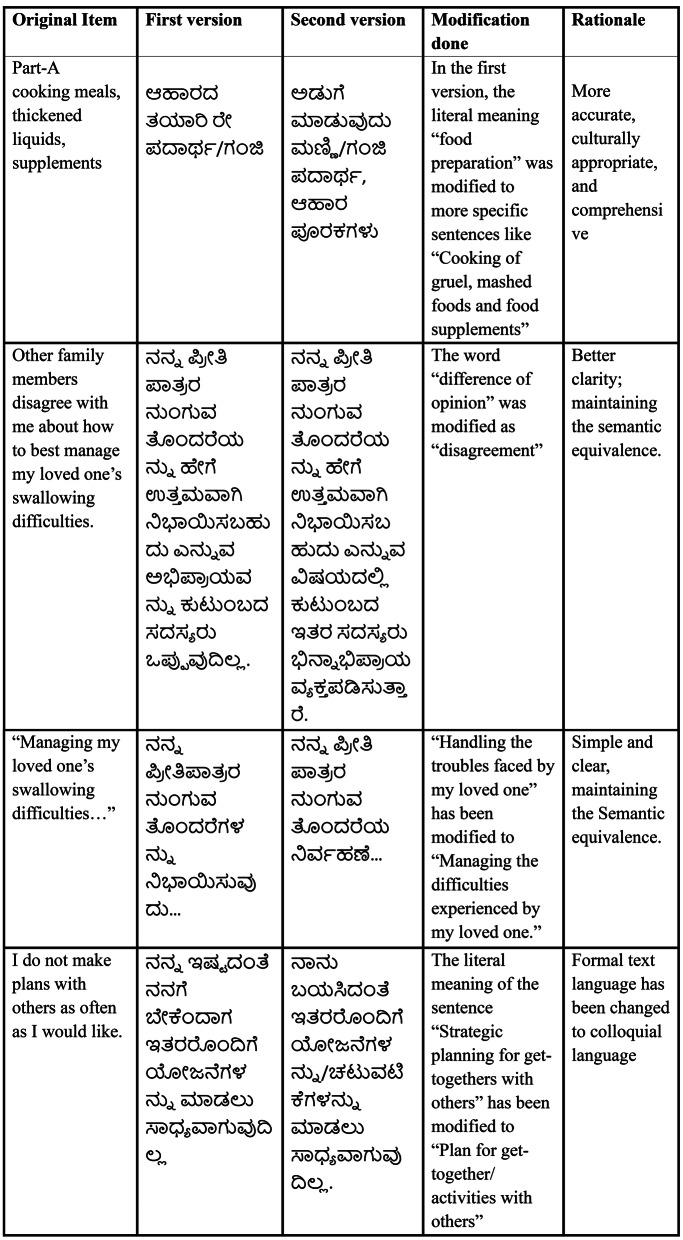

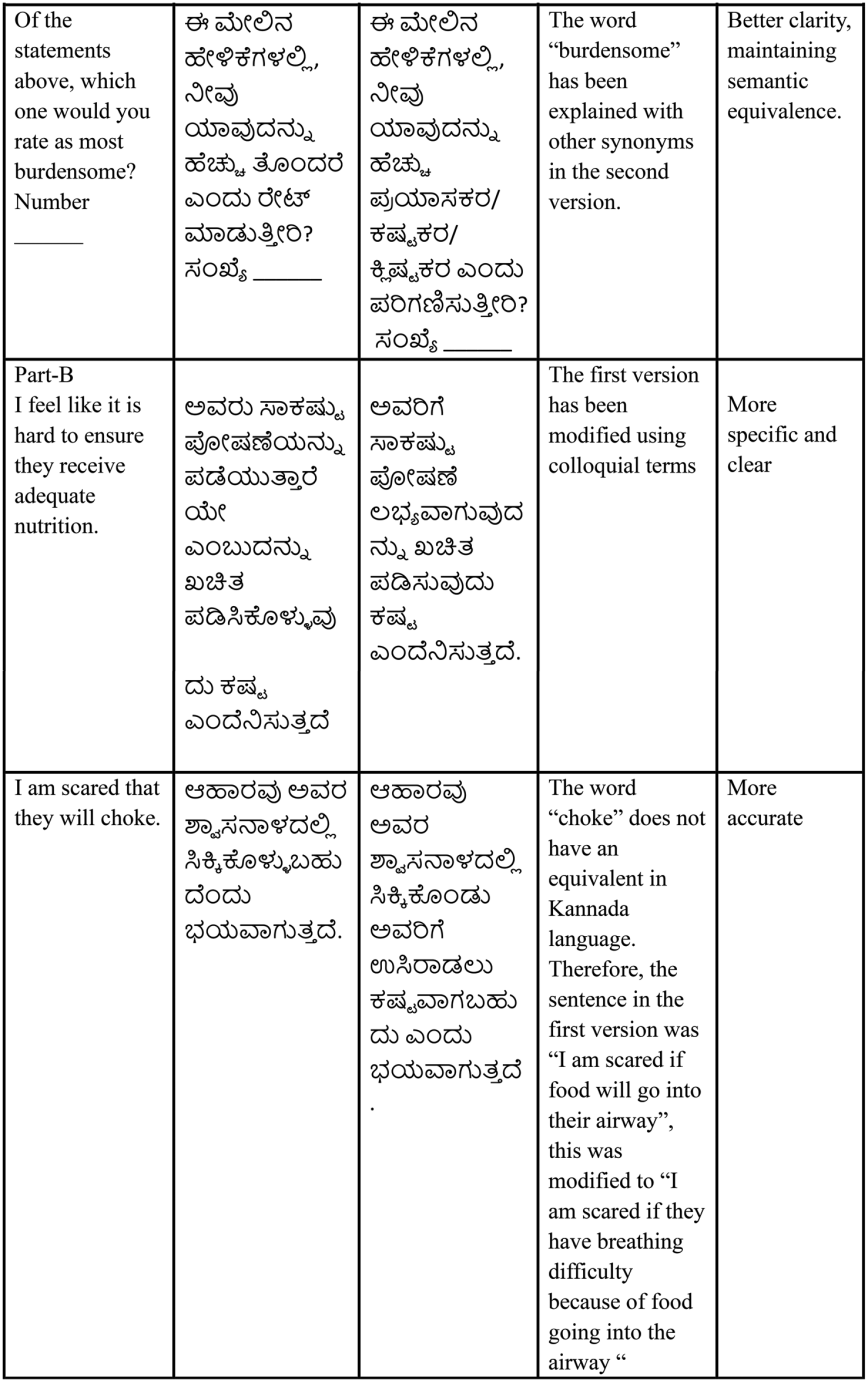
Modifications suggested during the cognitive debriefing process with professionals and caregivers

### Face validity assessment

All five caregivers reported that the tool was easy to understand, culturally appropriate, and relevant to their caregiving experiences, with no suggestions for modifications. This feedback confirmed that the items were clear, appropriately adapted, and suitable for the target population. As no changes were needed, the final version of the Kannada CARES tool was retained as initially adapted.

### Pre-testing

The study included 48 caregivers (18 to 68 years old) caring for adult dysphagia cancer survivors (definitive surgery, adjuvant radiation, definitive radiation, adjuvant chemoradiotherapy, definitive chemoradiotherapy, and palliative). The study findings are discussed on the basis of the responses obtained from primary caregivers of dysphagia patients via the Kannada version of the CARES screening tool.

#### Demographic characteristics

The caregivers were from a heterogeneous group with a mean age of 41.0 ± 13.3 years. Most caregivers were female spousal caregivers (*n* = 19; 39.6%). Almost 50% of the caregivers were unemployed and had an education qualification of 10th grade or less. Approximately 27.1% of them reported caring for their family members with dysphagia for more than 10 h daily (Table 3). The caregiver’s medical history was gathered using open-ended questions. Only the conditions reported by the caregiver are reported. In terms of care recipients (refer to Appendix B), male care recipients (77.1%) outnumbered female care recipients (22.9%). The tumor locations and types of treatment received varied widely, with buccal mucosa cancer being the most common (20.8%). Approximately 47.9% of the care recipients received treatment for stage IV cancer, and most received adjuvant radiotherapy (37.5%) as part of their treatment regimen.

**Table 3:** Demographic details of the caregivers

Demographic details	*N* = 48
Caregiver age (in years), *M ± SD*	41.0 ± 13.3 (range: 18–68)
Caregiver Sex Male Female	***n (%)*** 19 (39.6) 29 (60.4)
Caregiver’s relationship with care-recipients Spouse Child Grandchild Sibling In-law	22 (45.8) 18 (37.5) 2 (4.2) 2 (4.2) 4 (8.4)
Employment status Employed Unemployed	24 (50.0) 24 (50.0)
Education History ≤ 10th grade 12th grade Undergraduate Postgraduate	24 (50.0) 13 (27.1) 10 (20.8) 1 (2.1)
Hours of caregiving (hours/day) 2–3 3–4 4–5 5–6 6–10 More than 10 h	10 (20.8) 3 (6.30) 10 (20.8) 10 (22.8) 2 (4.40) 13 (27.10)
Family Dynamics Nuclear Joint	33 (68.8) 15 (31.3)
Medical History Hypertension Coronary Artery Diseases	3 (75.0) 1 (25.0)

Data are number (percentage) unless and otherwise specified

Nuclear: Parents and children only

Joint: Extended family members living together

#### Subscale scores and total scores of the Kannada version of the CARES screening tool

The mean and standard deviation (SD) score for Part A, the Checklist of Behavioral and Functional Changes, was 6.19 ± 2.57, with scores ranging from 1 to 10. For Part B, the Measures of Subjective Caregiver Stress, the mean and standard deviation (SD) were 7.94 ± 3.60, with scores ranging from 0 to 16. The overall total score for the Kannada CARES tool had a mean of 14.1 ± 5.44, with a minimum score of 3 and a maximum score of 26. Thus, indicating that the caregivers of individuals with dysphagia experienced significant behavioral and functional changes, leading to an increase in subjective caregiver stress [17].

#### List of statements rated most burdensome by the caregivers

**Fig. 2:**
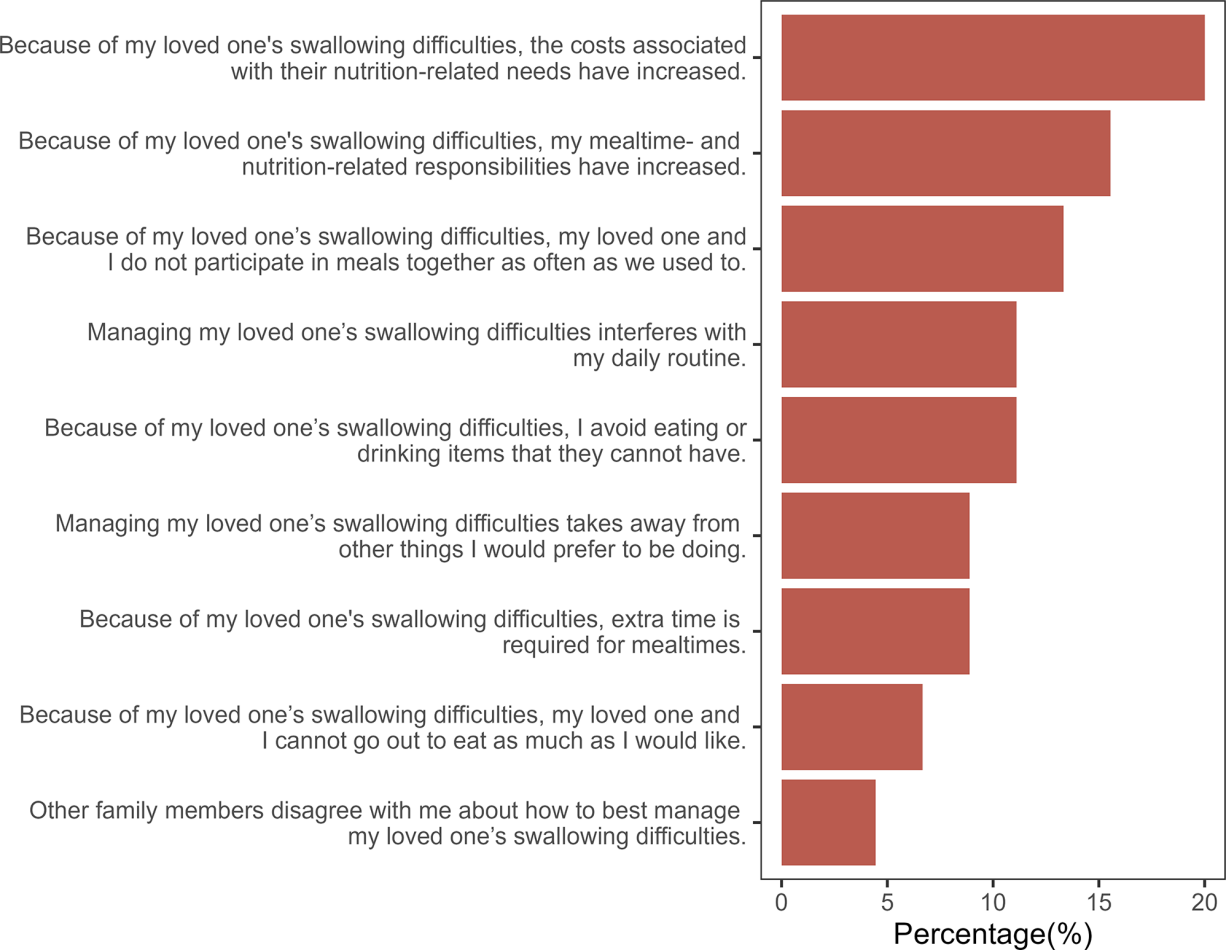
Statements in Part A rated as most burdensome by the caregivers

In Part A (functional and behavioral changes), approximately 20% of the caregivers rated the costs associated with the care recipient’s nutritional needs as the most burdensome among the 10 statements. Similarly, a higher number of caregivers reported an increase in meal-related responsibilities (15.55%) and a decrease in mealtime participation (13.33%) as the most burdensome (Fig. 2).

**Fig. 3:**
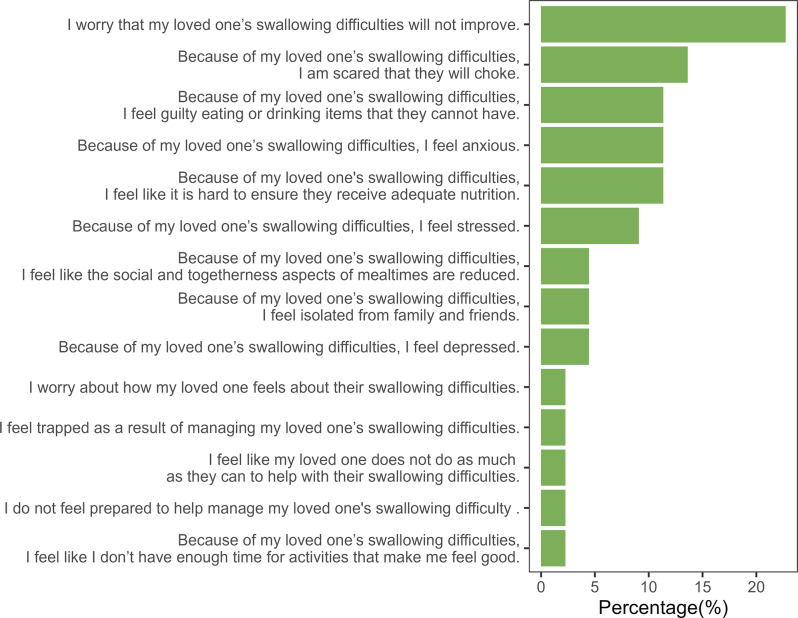
Statements in Part B rated as most burdensome by the caregivers

In Part B (Measures of Subjective Caregiver Stress), 22.72% of the caregivers reported that increasing subject stress was linked to their concerns about the prognosis of the care recipient’s swallowing difficulties. In addition, 13.63% of the caregivers indicated fears about their care recipient choking. Furthermore, 11.36% of the caregivers also reported feeling guilty and anxious due to their care recipient’s swallowing difficulty, thus posing challenges in ensuring adequate nutritional needs and contributing to their stress levels (Fig. 3). After completing each section, the caregivers were asked to identify the single most burdensome item by indicating its number.

#### Internal consistency

Cronbach’s alpha was used to measure the internal consistency of the Kannada CARES tool. The results revealed excellent internal consistency for the overall (α = 0.87) and individual domains of the Kannada CARES; Part A—Checklist of Behavioral and Functional Changes—and Part B—Measures of Subjective Caregiver Stress—were α = 0.76 and α = 0.85, respectively (Table 4). Furthermore, the item–total correlation of each item of the subscale was greater than 0.8, indicating that each item of the tool significantly contributed to the tool (Table 5) [21].

**Table 4:** Cronbach’s alpha for Part A—checklist of behavioral and functional changes, Part B—Measures of subjective caregiver stress and overall scale

Kannada CARES scales	No. of Items	Cronbach’s α result
Part A—Checklist of Behavioral and Functional Changes	10	0.76
Part B—Measures of Subjective Caregiver Stress	16	0.85
Total scale	26	0.87

**Table 5:** Intra-class correlation coefficient for each individual item

Kannada CARES subscales	Cronbach’s α result
**Part A—Checklist of Behavioral and Functional Changes**	
1 2 3 4 5 6 7 8 9 10	0.88 0.88 0.87 0.87 0.87 0.87 0.87 0.86 0.86 0.88
**Part B—Measures of Subjective Caregiver Stress**	
1 2 3 4 5 6 7 8 9 10 11 12 13 14 15 16	0.87 0.88 0.87 0.88 0.87 0.86 0.86 0.86 0.87 0.87 0.87 0.87 0.87 0.87 0.87 0.87

#### Correlation

The severity of dysphagia was assessed in all care recipients via the MASA-C scale, and the majority of care recipients had scores lower than 163 (81.3%), indicating severe dysphagia (refer to Appendix A). Pearson’s correlation coefficient was calculated to determine the relationship between clinician-rated MASA-C scores and the corresponding CARE score provided by their caregiver. A negative correlation between these two total scores, although not statistically significant (*r*= -0.260; *p* = 0.074), indicated that when the CARE score increased, the MASA-C score decreased.

## Discussion

Dysphagia is a complex disorder with multifaceted ramifications that significantly impact both individuals with dysphagia and their caregivers. The literature reports that dysphagia leads to caregiver burden, with 71% of caregivers experiencing the burden of one form or another [6, 22]. The physical and mental health of the caregiver could be an independent factor in determining the quality of care received by the care recipient [10, 23]. Given the significant burden experienced by caregivers, it is crucial to implement intervention strategies that consider both the health and well-being of caregivers and care recipients with dysphagia holistically [10]. Therefore, understanding the multitude of sequelae of dysphagia in caregivers and quantifying these consequences for caregivers’ everyday lives are key components in empowering caregivers.

Following the translation and adaptation of the English version of the CARES to the Kannada language, the reliability of the Kannada version of the CARES was assessed from the responses of 48 caregivers of individuals with dysphagia associated with head and neck cancer. A higher Cronbach’s alpha value (0.87) for the Kannada CARES indicates that the item in the Kannada tool measured the underlying construct. The Cronbach’s alpha value of the Kannada version of the CARES was found to be consistent and higher than that of the original English version of the CARES (0.79), indicating its reliability in assessing caregiver burden [17].

The mean scores obtained for Part A (6.19 **±** 2.57) and Part B (7.94 **±** 3.60) indicated a higher level of caregiver burden. This finding supports the notion of the original study that caregivers of dysphagic individuals experience caregiver burden and that the Kannada CARES tool can be used to identify caregiver burden. Furthermore, the scale was found to be helpful in identifying the most burdensome component experienced by caregivers. Under behavioral and functional changes (Part A), the costs associated with the care recipient’s nutritional needs, decreased mealtime participation, and increased meal-related responsibilities were reported as the most burdensome (Fig. 2). However, the stress associated with the prognosis of the care recipient’s swallowing difficulties, fear of choking, and feelings of guilt and anxiety were found to contribute to the perception of burden under measures of subjective caregiver stress (Part B) (Fig. 3). Additionally, the negative correlation between the MASA-C score and the CARES total score may be attributed to the relationship between these two tools. Higher MASA-C scores indicate better swallowing function, whereas higher CARES scores suggest a higher caregiver burden. However, as the correlation was not statistically significant, future work with a larger sample size may be needed to further test the relationship between these tools. Test-retest reliability data were collected for a subset of participants (*n* = 10). However, due to the small sample size, these results are not reported (for details, refer to Appendix C).

Hence, it can be said that the items in the CARES provide clear insight into the preparedness of caregivers and the stress associated with caring for individuals with dysphagia. By administering the Kannada CARES tool, SLPs will be well-equipped to address concerns and provide education and strategies to reduce the risk of choking. Additionally, on the basis of these responses, SLPs can provide referrals to other healthcare professionals to support and relieve caregiver burdens.

The current study has several limitations. First, the study focused on the linguistic and cultural adaptation of the CARES tool to Kannada. While the results suggest that the tool is linguistically equivalent and reliable, cross-cultural validation was not conducted. Hence, future research should include a comprehensive cross-cultural validation process to confirm the tool’s applicability across diverse Kannada-speaking populations. Second, the small sample size (*n* = 10) for test-retest reliability limits the generalizability of the findings. Finally, caregiving hours were collected as a continuous variable and later categorized for analysis. This categorization may limit the interpretation of actual caregiving time.

The study highlights the importance of using tools such as the Kannada CARES to identify caregiver burden in the context of dysphagia management. By understanding caregiver challenges, clinicians can provide targeted education and support to address identified burdens effectively. This may involve providing cost-saving strategies, ensuring caregiver training on safe swallowing and efficient feeding, and coaching to optimize mealtime engagement. However, SLPs should refer caregivers to integrated psychological services for emotional support related to prognosis, guilt, or anxiety, which falls outside the typical scope of SLPs. While exploring the roots of caregiver stress is within an SLP’s role, directly managing these emotional sequelae is not. Following the identification of stress sources, SLPs can assist in developing practical strategies for lowering burdens or providing recommendations for additional services such as social work or family therapy. Thus, the Kannada CARES tool can capture caregiver burden in dysphagia management for holistic care. The scale can also assess the impact of intervention strategies on improving caregiver burden. SLPs play a key role in recognizing caregiver stress factors and initiating referrals or recommendations to address modifiable sources of burden. An integrated, multidisciplinary approach is essential for fully supporting caregivers’ psychological and emotional needs.

## Conclusion

The current study involved cross-cultural translation and adaptation of the original CARES screening tool in the Kannada language. The findings shed light on the negative impact of dysphagia on caregivers, highlighting the need to address and alleviate their burden. To help mitigate the burden on caregivers of persons with dysphagia, SLPs must, in their management, incorporate educating and empowering caregivers in understandable ways to provide them with the means to recognize the impact of dysphagia to devise and optimize holistic management.

## Electronic supplementary material

Below is the link to the electronic supplementary material.

Supplementary Material 1

Supplementary Material 2

Supplementary Material 3

Supplementary Material 4

## Data Availability

The translated Kannada version of the Caregiver Analysis of Reported Experiences with Swallowing Disorders (CARES) Screening Tool is available as supplementary material. Other datasets generated and/or analyzed during the current study are available from the corresponding author upon reasonable request.
