# Supplementary material for: Cross-cultural adaptation, translation and pre-testing of the Caregiver Analysis of Reported Experiences with Swallowing Disorders (CARES) screening tool in Kannada
Source: J Patient Rep Outcomes. 2025 Sep 1;9:109. doi: 10.1186/s41687-025-00863-8 (PMC12401859; doi:10.1186/s41687-025-00863-8)
Supplement: Supplementary file 1 — Supplementary Material 1 [file 41687_2025_863_MOESM1_ESM.docx]

**Appendix D: CARES Kannada v.2 Jan 2023**

**ನುಂಗಲು ತೊಂದರೆ ಇರುವವರ ಆರೈಕೆದಾರರ (ಕೇರ್ಸ್) ಅನುಭವಗಳ ವರದಿಯ ವಿಶ್ಲೇಷಣೆ 2.1**

**ಭಾಗ ಎ: ವರ್ತನೆ ಮತ್ತು ಕ್ರಿಯಾತ್ಮಕ ಬದಲಾವಣೆಯ ಪರಿಶೀಲನಾ ಪಟ್ಟಿ**

ಕಳೆದ ತಿಂಗಳಿನಲ್ಲಿ ತಿನ್ನಲು ಅಥವಾ ನುಂಗಲು ತೊಂದರೆ ಅನುಭವಿಸಿದ ನಿಮ್ಮ ಪ್ರೀತಿ ಪಾತ್ರರು / ಆರೈಕೆ ಸ್ವೀಕರಿಸುವವರ ಬಗ್ಗೆ ಈ ಕೆಳಗಿನ ಪ್ರತಿಯೊಂದು ಹೇಳಿಕೆಗಳ ಕುರಿತು ನೀವು ಚೆನ್ನಾಗಿ ಆಲೋಚಿಸಿ. ಈ ಹೇಳಿಕೆಗಳಲ್ಲಿ ವಿವರಿಸಿದ ಪರಿಸ್ಥಿತಿಯು ನಿಮಗೆ ತೊಂದರೆಯನ್ನುಂಟುಮಾಡಿದೆಯೇ? ಅದು ಸಂಭವಿಸದಿದ್ದಲ್ಲಿ, ದಯವಿಟ್ಟು “ಅನ್ವಯಿಸುವುದಿಲ್ಲ” (N/A) ಎಂದು ಸೂಚಿಸಿ.

|  | ಕಳೆದ ತಿಂಗಳಲ್ಲಿ, ಈ ಪರಿಸ್ಥಿತಿಯು ನಿಮಗೆ ತೊಂದರೆಯನ್ನುಂಟುಮಾಡಿದೆಯೇ? |
| --- | --- |
| 1. ನನ್ನ ಪ್ರೀತಿ ಪಾತ್ರರ ನುಂಗುವ ತೊಂದರೆಯಿಂದಾಗಿ ಊಟದ ತಯಾರಿಗೆ ಹೆಚ್ಚು ಸಮಯ ಬೇಕಾಗುತ್ತದೆ (ಉದಾ: ಸೂಕ್ತವಾದ ಆಹಾರವನ್ನು ಹುಡುಕುವುದು, ಅಡುಗೆ ಮಾಡುವುದು, ಟ್ಯೂಬ್ ಮೂಲಕ ಆಹಾರ ನೀಡಲು ಸಿದ್ಧತೆ, ನನ್ನ ಪ್ರೀತಿ ಪ್ರಾತರು ಕುಡಿಯುವುದು/ತಿನ್ನುವುದನ್ನು ನೋಡುವುದು). | ಹೌದು ಇಲ್ಲ ಅನ್ವಯಿಸುವುದಿಲ್ಲ (N/A) |
| 2. ನನ್ನ ಪ್ರೀತಿ ಪಾತ್ರರ ನುಂಗುವ ತೊಂದರೆಯಿಂದಾಗಿ ಪೋಷಣೆ ಮತ್ತು ಊಟದ ಕುರಿತು ನನ್ನ ಜವಾಬ್ದಾರಿ ಹೆಚ್ಚಿದೆ (ಉದಾ: ಸಾಮಾನು ಖರೀದಿ, ಅಡುಗೆ, ಟ್ಯೂಬ್ ಫೀಡಿಂಗ್ಗೆ ಸಂಬಂಧಿತ). | ಹೌದು ಇಲ್ಲ ಅನ್ವಯಿಸುವುದಿಲ್ಲ (N/A) |
| 3. ನನ್ನ ಪ್ರೀತಿ ಪಾತ್ರರ ನುಂಗುವ ತೊಂದರೆಯಿಂದಾಗಿ ಅವರ ಪೌಷ್ಟಿಕ ಆಹಾರ ಕುರಿತ ಅಗತ್ಯಗಳು ಮತ್ತು ವೆಚ್ಚಗಳು ಹೆಚ್ಚಾಗಿವೆ (ಉದಾ: ಟ್ಯೂಬ್ ಮೂಲಕ ಆಹಾರ ನೀಡಲು ಬೇಕಾಗುವ ಸಲಕರಣೆಗಳು, ಮಣ್ಣಿ/ಗಂಜಿ ಪದಾರ್ಥ, ಆಹಾರ ಪೂರಕಗಳು). | ಹೌದು ಇಲ್ಲ ಅನ್ವಯಿಸುವುದಿಲ್ಲ (N/A) |
| 4. ನನ್ನ ಪ್ರೀತಿ ಪಾತ್ರರ ನುಂಗುವ ತೊಂದರೆಯನ್ನು ಹೇಗೆ ಉತ್ತಮವಾಗಿ ನಿಭಾಯಿಸಬಹುದು ಎನ್ನುವ ವಿಷಯದಲ್ಲಿ ಕುಟುಂಬದ ಇತರ ಸದಸ್ಯರು ಭಿನ್ನಾಭಿಪ್ರಾಯ ವ್ಯಕ್ತಪಡಿಸುತ್ತಾರೆ. | ಹೌದು ಇಲ್ಲ ಅನ್ವಯಿಸುವುದಿಲ್ಲ (N/A) |
|  | ಕಳೆದ ತಿಂಗಳಲ್ಲಿ, ಈ ಪರಿಸ್ಥಿತಿಯು ನಿಮಗೆ ತೊಂದರೆಯನ್ನುಂಟುಮಾಡಿದೆಯೇ? |
| 5. ನನ್ನ ಪ್ರೀತಿ ಪಾತ್ರರ ನುಂಗುವ ತೊಂದರೆಯ ನಿರ್ವಹಣೆ, ನನ್ನ ದಿನನಿತ್ಯದ ದಿನಚರಿ ನಿರ್ವಹಿಸಲು ಅಡ್ಡಿ ಮಾಡುತ್ತದೆ (ಉದಾ: ನೌಕರಿ, ಶಾಲಾ ಕೆಲಸ, ಮನೆ ಕೆಲಸ). | ಹೌದು ಇಲ್ಲ ಅನ್ವಯಿಸುವುದಿಲ್ಲ (N/A) |
| 6. ನನ್ನ ಪ್ರೀತಿ ಪಾತ್ರರ ನುಂಗುವ ತೊಂದರೆಗಳ ನಿರ್ವಹಣೆ ನನಗೆ ಇಷ್ಟವಿರುವ ಇತರ ಚಟುವಟಿಕೆಗಳಿಂದ ನನ್ನನ್ನು ದೂರವಿರಿಸುತ್ತದೆ (ಉದಾಹರಣೆಗೆ: ವಿರಾಮ ಚಟುವಟಿಕೆಗಳು). | ಹೌದು ಇಲ್ಲ ಅನ್ವಯಿಸುವುದಿಲ್ಲ (N/A) |
| 7. ನನ್ನ ಪ್ರೀತಿ ಪಾತ್ರರ ನುಂಗುವ ತೊಂದರೆಗಳ ಕಾರಣ, ನಾನು ಮತ್ತು ನನ್ನ ಪ್ರೀತಿ ಪಾತ್ರರು ಹಿಂದಿನಂತೆ ಒಟ್ಟಾಗಿ ಊಟ ಮಾಡಲಾಗುವುದಿಲ್ಲ. | ಹೌದು ಇಲ್ಲ ಅನ್ವಯಿಸುವುದಿಲ್ಲ (N/A) |
| 8. ನನ್ನ ಪ್ರೀತಿ ಪಾತ್ರರ ನುಂಗುವ ತೊಂದರೆಯಿಂದಾಗಿ ನಾನು ಬಯಸಿದಂತೆ ಇತರರೊಂದಿಗೆ ಯೋಜನೆಗಳನ್ನು/ ಚಟುವಟಿಕೆಗಳನ್ನು ಮಾಡಲು ಸಾಧ್ಯವಾಗುವುದಿಲ್ಲ. | ಹೌದು ಇಲ್ಲ ಅನ್ವಯಿಸುವುದಿಲ್ಲ (N/A) |
| 9. ನನ್ನ ಪ್ರೀತಿ ಪಾತ್ರರ ನುಂಗುವ ತೊಂದರೆಗಳಿಂದಾಗಿ, ನನ್ನ ಪ್ರೀತಿಪಾತ್ರರು ಮತ್ತು ನಾನು ನನಗೆ ಇಷ್ಟವಿರುವಂತೆ ಹೊರಗೆ ಹೋಗಿ ತಿನ್ನಲು ಸಾಧ್ಯವಾಗುವುದಿಲ್ಲ. | ಹೌದು ಇಲ್ಲ ಅನ್ವಯಿಸುವುದಿಲ್ಲ (N/A) |
| 10. ನನ್ನ ಪ್ರೀತಿಪಾತ್ರರ ನುಂಗುವ ತೊಂದರೆಗಳ ಕಾರಣ, ನಾನು ಅವರಿಗೆ ಸೇವಿಸಲು ಆಗದಂತಹ ಆಹಾರವನ್ನು ತಿನ್ನುವುದು ಅಥವಾ ಕುಡಿಯುವುದರಿಂದ ದೂರವಿರುತ್ತೇನೆ. | ಹೌದು ಇಲ್ಲ ಅನ್ವಯಿಸುವುದಿಲ್ಲ (N/A) |
| **ಈ ಮೇಲಿನ 10 ಹೇಳಿಕೆಗಳಲ್ಲಿ, ನೀವು ಯಾವುದನ್ನು ಹೆಚ್ಚು ಪ್ರಯಾಸಕರ/ ಕಷ್ಟಕರ/ ಕ್ಲಿಷ್ಟಕರ ಎಂದು ಪರಿಗಣಿಸುತ್ತೀರಿ? ಸಂಖ್ಯೆ ______** | |

**ಭಾಗ ಬಿ: ಆರೈಕೆದಾರರ ಒತ್ತಡದ ಕುರಿತು ವ್ಯಕ್ತಿನಿಷ್ಠ ಪರಿಶೀಲನಾ ಪಟ್ಟಿ**

ಈ ಕೆಳಗಿನ ಪ್ರತಿಯೊಂದು ಹೇಳಿಕೆಗಳನ್ನು ತಿನ್ನಲು ಅಥವಾ ನುಂಗಲು ತೊಂದರೆ ಇರುವ ನಿಮ್ಮ ಪ್ರೀತಿಪಾತ್ರರ/ಆರೈಕೆ ಸ್ವೀಕರಿಸುವವರ ಗಮನದಲ್ಲಿಟ್ಟುಕೊಂಡು ಚೆನ್ನಾಗಿ ಯೋಚಿಸಿ ಉತ್ತರಿಸಿ. ಈ ಹೇಳಿಕೆಗಳು ನಿಮ್ಮ ವಿಚಾರದಲ್ಲಿ ನಿಜ ಎಂದು ಅನಿಸಿದೆಯೇ?

|  | ಕಳೆದ ತಿಂಗಳಲ್ಲಿ, ಈ ಪರಿಸ್ಥಿತಿಯು ನಿಮಗೆ ತೊಂದರೆಯನ್ನುಂಟುಮಾಡಿದೆಯೇ? |
| --- | --- |
| 1. ನನ್ನ ಪ್ರೀತಿಪಾತ್ರರ ನುಂಗುವ ತೊಂದರೆಯನ್ನು ನಿರ್ವಹಿಸಲು ನಾನು ಸಜ್ಜಾಗಿಲ್ಲ ಎಂದು ಭಾವಿಸುತ್ತೇನೆ (ಉದಾ: ಟ್ಯೂಬ್ ಫೀಡಿಂಗ್, ಮಣ್ಣಿ/ಗಂಜಿ ಪದಾರ್ಥ). | ಹೌದು ಇಲ್ಲ ಅನ್ವಯಿಸುವುದಿಲ್ಲ (N/A) |
| 2. ನನ್ನ ಪ್ರೀತಿಪಾತ್ರರ ನುಂಗುವ ತೊಂದರೆಗಳಿಂದಾಗಿ, ಅವರಿಗೆ ಸಾಕಷ್ಟು ಪೋಷಣೆ ಲಭ್ಯವಾಗುವುದನ್ನು ಖಚಿತ ಪಡಿಸುವುದು ಕಷ್ಟ ಎಂದೆನಿಸುತ್ತದೆ. | ಹೌದು ಇಲ್ಲ ಅನ್ವಯಿಸುವುದಿಲ್ಲ (N/A) |
| 3. ನನ್ನ ಪ್ರೀತಿಪಾತ್ರರ ನುಂಗುವ ಸಮಸ್ಯೆಯ ಪರಿಹಾರಕ್ಕೆ ಸ್ವತಃ ಅವರೆಷ್ಟು ಪ್ರಯತ್ನಿಸಬೇಕೋ ಅಷ್ಟು ಅವರು ಪ್ರಯತ್ನಿಸುವುದಿಲ್ಲ ಎಂದು ನನಗನಿಸುತ್ತದೆ. | ಹೌದು ಇಲ್ಲ ಅನ್ವಯಿಸುವುದಿಲ್ಲ (N/A) |
| 4. ನನ್ನ ಪ್ರೀತಿ ಪಾತ್ರರ ನುಂಗುವ ತೊಂದರೆಗಳಿಂದಾಗಿ, ಆಹಾರವು ಅವರ ಶ್ವಾಸನಾಳದಲ್ಲಿ ಸಿಕ್ಕಿಕೊಂಡು ಅವರಿಗೆ ಉಸಿರಾಡಲು ಕಷ್ಟವಾಗಬಹುದು ಎಂದು ಭಯವಾಗುತ್ತದೆ. | ಹೌದು ಇಲ್ಲ ಅನ್ವಯಿಸುವುದಿಲ್ಲ (N/A) |
| 5. ನನ್ನ ಪ್ರೀತಿಪಾತ್ರರ ನುಂಗುವ ತೊಂದರೆಗಳಿಂದಾಗಿ, ಅವರು ತಿನ್ನಲು, ಕುಡಿಯಲು ಸಾಧ್ಯವಿಲ್ಲದ ವಸ್ತುಗಳನ್ನು ನಾನು ಸೇವಿಸುವುದು ನನಗೆ ತಪ್ಪಿತಸ್ತನೆಂಬ ಭಾವನೆ ಉಂಟು ಮಾಡುತ್ತದೆ. | ಹೌದು ಇಲ್ಲ ಅನ್ವಯಿಸುವುದಿಲ್ಲ (N/A) |
| 6. ನನ್ನ ಪ್ರೀತಿಪಾತ್ರರ ನುಂಗುವ ತೊಂದರೆಗಳಿಂದಾಗಿ, ನನ್ನ ವೈಯಕ್ತಿಕ, ದೈಹಿಕ ಆರೋಗ್ಯದ ಬಗ್ಗೆ ಗಮನ ಹರಿಸಲು ಸಾಕಷ್ಟು ಸಮಯವಿಲ್ಲ ಎಂದು ಭಾವಿಸುತ್ತೇನೆ. | ಹೌದು ಇಲ್ಲ ಅನ್ವಯಿಸುವುದಿಲ್ಲ (N/A) |
| 7. ನನ್ನ ಪ್ರೀತಿಪಾತ್ರರ ನುಂಗುವ ತೊಂದರೆಯಿಂದಾಗಿ ನನಗೆ ಖುಷಿ ಕೊಡುವ ಚಟುವಟಿಕೆಗಳಲ್ಲಿ ಪಾಲ್ಗೊಳ್ಳಲು ಸಮಯವಿಲ್ಲ ಎಂದು ಭಾವಿಸುತ್ತೇನೆ. | ಹೌದು ಇಲ್ಲ ಅನ್ವಯಿಸುವುದಿಲ್ಲ (N/A) |
| 8. ನನ್ನ ಪ್ರೀತಿಪಾತ್ರರಿಗೆ ನುಂಗುವ ತೊಂದರೆ ಇರುವುದರಿಂದ, ನಾನು ಖಿನ್ನತೆಗೊಳಗಾಗಿದ್ದೇನೆ ಎಂದೆನಿಸುತ್ತದೆ. | ಹೌದು ಇಲ್ಲ ಅನ್ವಯಿಸುವುದಿಲ್ಲ (N/A) |
|  | ಕಳೆದ ತಿಂಗಳಲ್ಲಿ, ಈ ಪರಿಸ್ಥಿತಿಯು ನಿಮಗೆ ತೊಂದರೆಯನ್ನುಂಟುಮಾಡಿದೆಯೇ? |
| 9. ನನ್ನ ಪ್ರೀತಿಪಾತ್ರರ ನುಂಗುವ ತೊಂದರೆಯಿಂದಾಗಿ ಒತ್ತಡಕ್ಕೊಳಗಾಗಿದ್ದೇನೆ ಎಂದೆನಿಸುತ್ತದೆ. | ಹೌದು ಇಲ್ಲ ಅನ್ವಯಿಸುವುದಿಲ್ಲ (N/A) |
| 10. ನನ್ನ ಪ್ರೀತಿಪಾತ್ರರ ನುಂಗುವ ತೊಂದರೆಯಿಂದಾಗಿ, ನಾನು ಆತಂಕವನ್ನು ಅನುಭವಿಸುತ್ತೇನೆ. | ಹೌದು ಇಲ್ಲ ಅನ್ವಯಿಸುವುದಿಲ್ಲ (N/A) |
| 11. ನನ್ನ ಪ್ರೀತಿಪಾತ್ರರು ನುಂಗುವ ತೊಂದರೆ ಹೊಂದಿರುವುದರಿಂದ ಇತರ ಜನರಿರುವಾಗ ನಾನು ಮುಜಗರ ಅನುಭವಿಸುತ್ತೇನೆ. | ಹೌದು ಇಲ್ಲ ಅನ್ವಯಿಸುವುದಿಲ್ಲ (N/A) |
| 12. ತಮ್ಮ ನುಂಗುವ ತೊಂದರೆಯ ಬಗ್ಗೆ ನನ್ನ ಪ್ರೀತಿ ಪಾತ್ರರು ಹೇಗೆ ಭಾವಿಸುತ್ತಾರೋ ಎಂದು ನನಗೆ ಚಿಂತೆಯಾಗುತ್ತದೆ. | ಹೌದು ಇಲ್ಲ ಅನ್ವಯಿಸುವುದಿಲ್ಲ (N/A) |
| 13. ನನ್ನ ಪ್ರೀತಿ ಪಾತ್ರರ ನುಂಗುವ ತೊಂದರೆಗಳ ಕಾರಣ, ಸಾಮಾಜಿಕವಾಗಿ ಮತ್ತು ಎಲ್ಲರ ಜೊತೆಗೂಡಿ ಊಟ ಮಾಡುವುದು ಕಡಿಮೆಯಾಗಿದೆ. | ಹೌದು ಇಲ್ಲ ಅನ್ವಯಿಸುವುದಿಲ್ಲ (N/A) |
| 14. ನನ್ನ ಪ್ರೀತಿಪಾತ್ರರ ನುಂಗುವ ತೊಂದರೆಗಳ ಕಾರಣ, ನಾನು ಕುಟುಂಬ ಮತ್ತು ಸ್ನೇಹಿತರಿಂದ ಪ್ರತ್ಯೇಕಿಸಲ್ಪಟ್ಟಂತೆ ಅನಿಸುತ್ತದೆ. | ಹೌದು ಇಲ್ಲ ಅನ್ವಯಿಸುವುದಿಲ್ಲ (N/A) |
| 15. ನನ್ನ ಪ್ರೀತಿಪಾತ್ರರ ನುಂಗುವ ತೊಂದರೆಗಳನ್ನು ನಿರ್ವಹಿಸಬೇಕಾದ ಪರಿಣಾಮವಾಗಿ ನಾನು ಸಿಕ್ಕಿಬಿದ್ದಂತೆ ಅನಿಸುತ್ತದೆ. | ಹೌದು ಇಲ್ಲ ಅನ್ವಯಿಸುವುದಿಲ್ಲ (N/A) |
| 16. ನನ್ನ ಪ್ರೀತಿಪಾತ್ರರ ನುಂಗುವ ತೊಂದರೆಗಳು ಸುಧಾರಿಸುವುದಿಲ್ಲ ಎಂದು ನಾನು ಚಿಂತಿಸುತ್ತೇನೆ. | ಹೌದು ಇಲ್ಲ ಅನ್ವಯಿಸುವುದಿಲ್ಲ (N/A) |
| **ಈ ಮೇಲಿನ 16 ಹೇಳಿಕೆಗಳಲ್ಲಿ, ನೀವು ಯಾವುದನ್ನು ಹೆಚ್ಚು ಪ್ರಯಾಸಕರ/ ಕಷ್ಟಕರ/ ಕ್ಲಿಷ್ಟಕರ ಎಂದು ಪರಿಗಣಿಸುತ್ತೀರಿ? ಸಂಖ್ಯೆ ______** | |
