# Supplementary material for: Cross-cultural adaptation, translation and pre-testing of the Caregiver Analysis of Reported Experiences with Swallowing Disorders (CARES) screening tool in Kannada
Source: J Patient Rep Outcomes. 2025 Sep 1;9:109. doi: 10.1186/s41687-025-00863-8 (PMC12401859; doi:10.1186/s41687-025-00863-8)
Supplement: Supplementary file 2 — Supplementary Material 2 [file 41687_2025_863_MOESM2_ESM.docx]

**Data collection form**

Demographic details

Subject Number:

**Caregiver information**

Age/sex:

Relationship with the care recipients:

Prior or current employment status:

Education History:

Hours of caregiving provided:

Family dynamics:

Caregiver's medical history (if any):

Phone number:

Address:

**Care recipient information**

Age/sex:

Primary site and stage of head and neck cancer:

Type of treatment:

Recommendation for diet modification; on NG feed or oral feed:
