# Supplementary material for: Cross-cultural adaptation, translation and pre-testing of the Caregiver Analysis of Reported Experiences with Swallowing Disorders (CARES) screening tool in Kannada
Source: J Patient Rep Outcomes. 2025 Sep 1;9:109. doi: 10.1186/s41687-025-00863-8 (PMC12401859; doi:10.1186/s41687-025-00863-8)
Supplement: Supplementary file 3 — Supplementary Material 3 [file 41687_2025_863_MOESM3_ESM.docx]

***Demographic details of the care recipients***

| Demographic details | N = 48 |
| --- | --- |
| Care-recipients age (in years), *M ± SD* | 58.9 ± 12.2 (range: 38 – 87) |
| Sex  Male  Female | ***n (%)***  37 (77.1)  11 (22.9) |
| Primary site of tumor*)*  Buccal mucosa  Tongue  Floor of mouth (FOM)  Retromolar Trigone (RTM)  Alveolus  Gingivobuccal sulcus, palate, and maxilla  Oropharynx  Hypopharynx  Pyriform sinus  Larynx  Supra-glottis  Glottis  Postcricoid | 10 (20.8)  7 (14.6)  4 (8.3)  5 (10.4)  2 (4.2)  2 (4.2)  2 (4.2)  6 (12.5)  2 (4.2)  2 (4.2)  3 (6.3)  1 (2.1)  2 (4.2) |
| Stage of cancer  Stage I  Stage II  Stage III  Stage IV | 2 (4.2)  10 (20.8)  13 (27.1)  23 (47.9) |
| Type of Treatment  Definitive Surgery  Definitive RT  Definitive CT-RT  Adjuvant RT  Adjuvant CT-RT  Palliative | 3 (6.3)  7 (14.6)  6 (12.5)  18 (37.5)  9 (18.8)  5 (10.4) |
| Type of feeding, *n (%)*  Tube feeding  Oral feeding | 23 (47.9)  25 (52.1) |
| Suggested oral diet modifications, *n (%)*  Pureed diet  Liquid diet | 20 (76.9)  6 (23.1) |
| MASA-C Scores, *n (%)*  200 – 184  183 – 174  173 – 164  ≤ 163 | 1 (2.1)  4 (8.3)  4 (8.3)  39 (81.3) |
| *Data are number (percentage) unless otherwise specified.*  *Note:* MASA-C = Mann’s Assessment of Swallowing Ability – Cancer; RT = Radiotherapy; CT- Chemotherapy | |
