# Supplementary material for: Cross-cultural adaptation, translation and pre-testing of the Caregiver Analysis of Reported Experiences with Swallowing Disorders (CARES) screening tool in Kannada
Source: J Patient Rep Outcomes. 2025 Sep 1;9:109. doi: 10.1186/s41687-025-00863-8 (PMC12401859; doi:10.1186/s41687-025-00863-8)
Supplement: Supplementary file 4 — Supplementary Material 4 [file 41687_2025_863_MOESM4_ESM.docx]

***Test-retest reliability***

## **Intra-class correlation for caregivers of dysphagia patients for test re-test reliability**

| **Kannada CARES scales** | **No. of Items** | **Cronbach’s α result** |
| --- | --- | --- |
| Part A—Checklist of Behavioral and Functional Changes | 10 | 0.87 |
| Part B—Measures of Subjective Caregiver Stress | 16 | 0.84 |
| Total scale | 26 | 0.89 |
